# Smartphone-Based Ecological Momentary Assessment to Monitor Opioid Use and Overdose Among People Who Use Opioids: Prospective Observational Feasibility Study

**DOI:** 10.2196/95655

**Published:** 2026-06-29

**Authors:** Kamal Gautam, Kiran Paudel, Jeffrey A Wickersham, Antoine Khati, Anushka Thapa, Sandesh Bhusal, Md Safaet Hossain Sujan, Sherry Pagoto, Toan Ha, Roman Shrestha

**Affiliations:** 1 Department of Allied Health Sciences, University of Connecticut, 358 Mansfield Road, Unit 1101, Storrs, CT, 06269-1101, United States, 1 860-486-2834; 2 Section of Infectious Diseases, Department of Medicine, Yale School of Medicine, Yale University, New Haven, CT, United States; 3 Department of Infectious Diseases and Microbiology, School of Public Health, University of Pittsburgh, Pittsburgh, PA, United States

**Keywords:** ecological momentary assessment, opioid overdose, naloxone, harm reduction, syringe service programs, substance use, mobile health

## Abstract

**Background:**

Opioids account for 76% of drug overdose deaths in the United States, with nearly 80,000 opioid overdose deaths annually. The risk of overdose is dynamic and influenced by rapidly changing behaviors and contexts that are not well captured by retrospective or infrequent assessments. Ecological momentary assessment (EMA) allows repeated near real-time reporting of behaviors and experiences in natural settings.

**Objective:**

This study evaluated the feasibility and acceptability of smartphone-based EMA among people who use opioids to monitor opioid use, overdose experiences, and naloxone access.

**Methods:**

Participants were recruited through the New Haven Syringe Services Program in New Haven, Connecticut, and completed fixed-time, twice-daily EMA prompts for 30 days using a smartphone app. EMA measures assessed behaviors and experiences occurring within the previous 12 hours, including opioid craving, plans to use drugs, opioid use, overdose experiences, and naloxone access. Feasibility was assessed through recruitment, retention, and EMA completion rates, as well as the reporting of drug use-related behaviors. Acceptability was evaluated using exit surveys assessing ease of use, burden, and privacy, as well as qualitative interviews.

**Results:**

Of the 13 screened individuals, 10 were enrolled and completed the baseline assessment. A total of 9 (90%) participants completed EMA and responded to 411 prompts, with an overall compliance rate of a mean of 85.0% (SD 8.7%). Compliance was similar across morning (84.2%, SD 13.1%) and evening (85.8%, SD 11.2%) prompts. Participants reported opioid cravings (274 reports), plans to use illicit drugs (252 reports), and opioid use (411 reports) during the study period. Two participants reported overdose events (5 reports) and were carrying naloxone during each overdose event. Acceptability ratings were high: all participants reported that EMA prompts were easy to understand and private, and most did not find them burdensome. Qualitative feedback further highlighted the ease of use, integration into daily routines, and increased self-awareness, although some participants reported emotional discomfort when reflecting on their substance use.

**Conclusions:**

Smartphone-based EMA was feasible and acceptable among people who use opioids recruited through a syringe service program. EMA may support the monitoring of opioid use, overdose experiences, and naloxone access, and it may inform future digital health interventions aimed at reducing harm, including overdose risk, among people who use opioids.

## Introduction

Opioid overdose remains a major public health challenge in the United States, with a high overdose mortality rate [1,2]. In 2023, more than 72,000 opioid-involved overdose deaths were reported, largely involving illegally manufactured fentanyl [3], and an estimated 5.6 million individuals aged 12 years or older met the criteria for opioid use disorder [4]. Although evidence-based treatments and harm-reduction strategies, such as expanded access to medications for opioid use disorder and syringe service programs (SSPs), have expanded [5,6], overdose risk remains high.

People who use opioids often experience fluctuations in drug use, craving, affective states, and contextual exposures, which can influence when and how overdoses occur [7,8]. Prior research has identified factors such as polysubstance use, reduced tolerance, environmental context, and the need for assistance with injections as contributors to overdose risk. Structural and psychosocial vulnerabilities, including homelessness, incarceration, unemployment, psychiatric comorbidity, and suicidal ideation or behavior, have also been linked to increased overdose risk [9,10]. However, most evidence has relied on retrospective self-reports or infrequent clinical assessments [9,11]. These approaches have limited ability to capture the dynamic and context-dependent nature of overdose risk in daily life among people who use opioids, many of whom experience unstable housing [12], unemployment [13], and inconsistent engagement with health care, including early treatment drop-out [14,15]. As a result, clinic-based assessments may not adequately reflect the dynamic conditions and circumstances under which opioid use and overdose events occur.

Ecological momentary assessment (EMA) provides one approach to address this limitation [16,17]. EMA involves collecting repeated self-reports of behaviors and experiences over short time intervals in natural settings as individuals go about their daily lives, thereby reducing recall bias and improving ecological validity [18,19]. Previous research has shown high levels of smartphone access, device ownership, and digital literacy among people who use opioids, including individuals experiencing housing instability, and that EMA has been successfully implemented among these populations [18,20-22]. However, existing EMA studies among this population have primarily focused on momentary predictors of drug use, such as craving, mood, and stress [17,18,23]. Less attention has been given to the use of EMA to monitor overdose-related experiences and naloxone availability and use, and gaps remain regarding the feasibility and acceptability of EMA protocols in populations facing significant social and structural vulnerabilities, such as people who use opioids.

To address these gaps, this pilot study evaluated the feasibility and acceptability of a smartphone-based EMA protocol for people who use opioids for monitoring opioid use, overdose experiences, and naloxone access in daily life. Feasibility was assessed through recruitment, retention, and EMA completion rates, whereas acceptability was assessed through poststudy surveys and qualitative exit interviews. The EMA protocol was designed to capture day-to-day opioid-related experiences and behaviors, including craving, opioid use, overdose experiences, and naloxone access and use through fixed twice-daily EMA prompts. The findings of this study are intended to inform the design and implementation of future EMA-based research and digital interventions targeting opioid use and overdose risk.

## Methods

### Study Design and Setting

The Smartphone Health Innovation Program was a prospective, observational pilot study that evaluated the feasibility and acceptability of repeated smartphone-based EMA among people who use opioids. The study was conducted in New Haven, Connecticut, at the New Haven Syringe Services Program (NHSSP). The NHSSP is a community harm-reduction program that provides sterile syringe exchange, hygiene supplies, overdose-prevention education, naloxone distribution, and referrals to medical and substance-use treatment services for people who use opioids. Participants were followed for 30 days and completed an in-person baseline visit, a 30-day EMA monitoring period, a structured support check-in approximately 7 days after enrollment, and an end-of-study visit with a qualitative interview.

### Recruitment, Eligibility, and Enrollment

Participants were recruited using convenience sampling through flyers posted at the NHSSP and surrounding community locations, word-of-mouth referrals, and referrals from local service organizations in the New Haven area (including treatment and harm-reduction programs, such as the APT Foundation, Inc). Potential participants either approached research staff while visiting the NHSSP for services (eg, syringe exchange visits) or contacted the study team by phone. Eligibility screening was conducted in person at the NHSSP or by telephone. Participants were eligible if they met the following criteria: (1) were aged 18 years or older, (2) reported use of at least one illicit opioid (eg, heroin and fentanyl) within the past 30 days, (3) owned a personal smartphone compatible with the study app (Android or iOS), and (4) resided in the Greater New Haven area.

Eligible participants attended an in-person enrollment visit at the NHSSP. Trained research assistants confirmed eligibility and obtained written informed consent in a private office. Participants then completed baseline questionnaires and onboarding procedures during the same visit. The enrollment and onboarding visit lasted approximately 30 minutes. Baseline questionnaires were administered electronically on a tablet and were primarily self-administered. Research staff read questions aloud and assisted participants when literacy or comprehension barriers were present. Participants were enrolled between November 2024 and February 2025. They completed 30 days of EMA monitoring following enrollment, and end-of-study assessments were conducted between January 2025 and March 2025.

### Study Procedures

#### EMA Protocol Development

EMA prompts were developed based on prior EMA research among people who use substances and were informed by the existing literature on overdose, opioid use, and harm-reduction behaviors [18,23]. The study team and community partners reviewed the prompts to ensure their relevance and appropriateness for the study population and setting.

#### EMA Monitoring

EMA was conducted using the mEMA app from Ilumivu, a platform designed for smartphone-based EMA studies. Participants received 2 fixed-time-based EMA prompts per day between 9 AM and 10 PM for 30 consecutive days (60 scheduled assessments) on their smartphones. Participants selected their preferred morning and evening times. The prompts were scheduled approximately 12 hours apart to capture nonoverlapping recall periods. This design was selected to support feasibility and engagement by reducing participant burden and allowing integration of EMA into their daily routines [24]. Each EMA remained active for 120 minutes. If unanswered, automated reminders were sent every 30 minutes (a maximum of 4 reminders) for each survey. After 120 minutes, the prompts expired. EMA prompts were brief and required approximately 1 to 2 minutes to complete. Research staff periodically reviewed incoming responses to ensure that prompts were received and completed and to troubleshoot technical problems with participants.

#### Day-7 Support Contact

Approximately 7 days after enrollment, the research staff conducted structured telephone check-ins. The staff checked on the participants’ safety and well-being, asked whether they were receiving notifications, experiencing difficulty charging their phones, or having problems answering EMAs, and reminded them to keep their phones charged and respond to EMAs promptly.

#### End-of-Study Visit

After 30 days, participants returned to the NHSSP to complete a poststudy questionnaire and a qualitative exit interview describing their experiences using the app to respond to the daily EMA prompts.

### Study Measures

#### EMA Measures

EMA items assessed recent behaviors, risk exposures, and internal states over the prior 12 hours. Participants reported substance use in response to the question, “In the last 12 hours, have you used any drug(s), legal or illegal, that are not prescribed to you?” If the answer was “yes,” participants selected the specific drugs used. Additional “yes” or “no” items assessed drug craving (“In the last 12 hours, did you experience any cravings for drug(s)?”), anticipated use (“Do you plan to use any drug(s) in the next 12 hours?”), sexual activity (“In the last 12 hours, have you had vaginal or anal sex with another person?”), overdose exposure (“In the past 12 hours, did you overdose on any drug(s)?” and “In the past 12 hours, did you witness or see another person overdose on drug(s)?”), and naloxone access (“Do you currently have Narcan available to you or someone else, if needed?”). All responses were coded as binary indicators for each EMA item.

Participants also rated affective and physical states using 0-10 numeric rating scales, including happiness (“In the last 12 hours, how happy were you?”), stress (“In the last 12 hours, how stressed were you?”), and physical pain (*“*In the last 12 hours, how intense was your physical pain?”). Higher scores indicated greater intensity of the respective state.

#### Baseline Measures

##### Sociodemographic Characteristics

Participants reported their age, sex, race or ethnicity, education, relationship status, employment status, monthly income, and current housing status.

##### Substance Use and Overdose History

Participants reported drug use in the past 12 months and the past 30 days, as well as the frequency of opioid use in the past 30 days. The route of administration was categorized as injection, smoking, snorting, oral use, or other. Lifetime overdose was assessed using a structured question that asked whether the participant had ever experienced an opioid overdose. The questionnaire provided symptom examples, including slowed or stopped breathing, blue lips or fingernails, unconsciousness, and inability to talk, to standardize interpretation. Participants also reported past 30-day and lifetime overdoses, as well as whether they had ever witnessed others overdose.

##### Substance Use Treatment Engagement

Participants were asked whether they were currently enrolled in a treatment program for opioid dependence (methadone, buprenorphine, extended-release naltrexone, or other), whether they had sought treatment in the past 12 months, and whether they had seen a substance use treatment provider in the past 6 months.

##### Mental Health

Depressive symptoms were measured using the Patient Health Questionnaire-2 [25], a brief, validated screener for depressive symptoms that has been used in drug and alcohol treatment populations [26]. It assesses *how often over the past 2 weeks participants were bothered by little interest or pleasure in doing things* and *feeling down, depressed, or hopeless*. Scores range from 0 to 6, with a cutoff score of ≥3 indicating moderate-to-severe depressive symptoms (Cronbach *α*=0.83).

Anxiety symptoms were measured using the Generalized Anxiety Disorder 7-item [27], a validated measure of anxiety symptoms that has been used in substance-using populations [28]. The Generalized Anxiety Disorder 7-item assesses how often over the past 2 weeks participants were bothered by 7 symptoms: feeling nervous, anxious, or on edge; not being able to stop or control worrying; worrying too much about different things; trouble relaxing; being so restless that it is hard to sit still; becoming easily annoyed or irritable; and feeling afraid as if something awful might happen. Scores range from 0 to 21, with scores of 10 or more indicating moderate-to-severe anxiety symptoms (Cronbach *α*=0.94).

Suicidal thoughts and behaviors were assessed using the suicidality module of the World Mental Health Composite International Diagnostic Interview. Participants reported lifetime suicidal ideation, suicide plans, and suicide attempts [29]. Participants also reported whether they had recent contact with a mental health provider (yes or no items). Current pain and stress levels were assessed using a visual analog scale ranging from 0 to 100, with higher scores indicating greater perceived pain or stress.

##### Digital Health Literacy

Digital health literacy was assessed using the 3-item Digital Health Care Literacy Scale [30]. Participants rated their agreement with statements indicating that they could independently: (1) use videoconferencing applications, (2) set up a video chat, and (3) solve basic technical problems. Responses were rated on a 5-point Likert agreement scale, ranging from “strongly disagree” to “strongly agree,” with a total score ranging from 0 to 12.

##### Feasibility and Acceptability Outcomes

Feasibility outcomes included recruitment, retention, and EMA compliance. Retention was defined as the completion of the end-of-study visit. EMA compliance was calculated as the proportion of completed prompts out of the total number of expected prompts among participants who initiated EMA monitoring. Morning and evening compliance were calculated separately, and to examine sustained engagement, completion rates were also summarized across 4 weekly intervals (days 1-7, 8-14, 15-21, and 22-30). Consistent with prior EMA literature among people who use substances, compliance rates of approximately 70% to 80% were considered indicative of acceptable feasibility [23,31-33].

Acceptability was assessed using a structured poststudy questionnaire. Participants rated statements such as “The daily EMA questions were easy to understand,” “I had privacy and confidentiality while answering the EMA prompts,” “The number of daily EMA was burdensome,” and their willingness to participate again using multilevel agreement scales. Participants also reported the emotional impact (positive, neutral, or negative), the convenience of EMA timing, the reliability of notifications, the helpfulness of research team reminders, and barriers to responding, including being busy, sleeping, missing notifications, not having a phone, or experiencing technical problems.

Qualitative exit interviews further explored participants’ experiences and perceived barriers to daily monitoring. The interview guide was developed by the study team based on the study objectives and prior EMA literature [34]. Participants were asked about their overall experiences and motivations for completing the daily EMA prompts, including perceived benefits and challenges, barriers to responding to prompts, and emotional reactions to questions about drug use (see Multimedia Appendix 1 for the exit interview guide).

### Data Analysis

Quantitative data were analyzed using Python version 3.13.4 (Python Software Foundation). Participant characteristics and feasibility outcomes were summarized using descriptive statistics. Continuous variables were summarized using means and SDs, and categorical variables were summarized using counts and percentages. EMA adherence was calculated at the participant level, separately for morning and evening prompts. To assess changes in engagement over time, EMA prompt completion was aggregated into 4 weekly intervals and summarized across participants. EMA behavioral responses (drug use, craving, planned use, overdose events, and naloxone access) were summarized as counts of endorsed events across completed observations. Data management and statistical summaries were conducted using the *pandas* and *NumPy* libraries, and figures were generated using *Matplotlib*.

Qualitative interviews were audio-recorded and transcribed verbatim. Data were analyzed using inductive thematic analysis, following the steps described by Braun and Clarke [35]. Interview transcripts were reviewed multiple times to become familiar with the data. Two coders independently conducted open coding of all transcripts and met regularly to review and refine the coding framework. Any discrepancies were resolved through discussion with the senior coder (KG), and codes were gradually grouped into broader thematic categories. Consistent with recommendations for enhancing rigor in qualitative research, coding decisions and thematic interpretations were reviewed collaboratively throughout the analytic process [36]. Qualitative data were managed and analyzed using Dedoose version 9.0.54 (SocioCultural Research Consultants, LLC).

### Ethical Considerations

The study was approved by the institutional review boards (IRB protocol #2000036555) at Yale University and the University of Connecticut. All participants provided written informed consent prior to participation. Participants received US $25 for completing the baseline visit and US $25 for completing the end-of-study visit and interview. EMA completion was incentivized on a per-prompt basis. Participants received US $1 for each completed EMA prompt (maximum of 60 prompts). The maximum total compensation per participant was US $110 over the 30-day study period.

## Results

### Feasibility

#### Recruitment and Participation

Thirteen individuals were assessed for eligibility through NHSSP, of whom 10 provided informed consent, were enrolled, and completed the baseline assessment and onboarding for the EMA app. Three eligible individuals did not proceed to enrollment because they did not attend their scheduled baseline interviews and were subsequently unreachable. One enrolled participant withdrew due to a scheduled cataract surgery before completing any EMA prompts. The remaining 9 participants completed the EMA prompts during the 30-day study period. EMA participation remained relatively stable across the study period, with 8 of the 9 participants continuing to respond to EMA prompts through week 4. Seven participants completed the exit survey and qualitative interviews following 30 days of EMA participation.

#### Participant Characteristics

The mean age of the 10 participants was 43.0 (SD 7.1) years (Table 1). Half identified as male, 4 (40%) as female, and 1 (10%) as transgender. Most participants had a high school education or lower (n=9, 90%), were currently unemployed, and 4 (40%) reported experiencing homelessness. Additionally, 9 (90%) participants reported injection drug use, and 8 (80%) reported daily opioid use in the past 30 days. Furthermore, 7 (70%) participants were receiving medications for opioid use disorder, including methadone maintenance treatment (n=5, 50%) and Vivitrol or other treatment programs (n=2, 20%). A history of opioid overdose was reported by 7 (70%) participants, and most had witnessed an overdose (n=9, 90%) and reported carrying naloxone (n=9, 90%). Digital health literacy was high (mean 10.3, SD 1.94; range 1‐12).

**Table 1.** Demographic characteristics (N=10).

Variable	Values
Age (y), mean (SD)	43.0 (7.1)
Gender, n (%)	
Women	4 (40)
Men	5 (50)
Transgender	1 (10)
Sexual orientation, n (%)	
Gay, lesbian, or bisexual	5 (50)
Straight or heterosexual	5 (50)
Ethnicity, n (%)	
White	5 (50)
Racial or ethnic minority	5 (50)
Current relationship status, n (%)	
Single	4 (40)
With partner	6 (60)
Education, n (%)	
High school or lower	9 (90)
Some colleges or higher	1 (10)
Currently unemployed, n (%)	10 (100)
Currently experiencing homelessness, n (%)	4 (40)
Smartphone type, n (%)	
Apple	2 (20)
Android	8 (80)
Digital health literacy, mean (SD)	10.3 (1.9)
Substance use and overdose history, n (%)	
Currently in MOUD^a^	7 (70)
Injected any drug (past 30 d)	9 (90)
Daily opioid use (past 30 d)	8 (80)
Ever overdosed with opioid	7 (70)
Opioid overdose (past 30 d)	1 (10)
Ever witnessed an overdose	9 (90)
Currently use Naloxone	9 (90)
Physical and psychological symptoms	
Pain, mean (SD)	70.5 (24.5)
Stressed, mean (SD)	75.3 (24.2)
Moderate to severe depressive symptoms, n (%)	4 (40)
Moderate to severe anxiety symptoms, n (%)	3 (30)
Suicidality history (ever), n (%)	
Suicidal thoughts	4 (40)
Suicidal plan	1 (10)
Suicidal attempt	3 (30)
Mental health services use (past 6 mo), n (%)	
Yes	6 (60)
No, but needed to	3 (30)
No, I did not need to	1 (10)

a MOUD: medications for opioid use disorder.

#### EMA Compliance

Among the 9 participants who completed at least 1 EMA, 411 out of 540 expected EMA prompts were completed during the study period. Overall, EMA compliance was high, with an average of 85.0% (SD 8.7%). Compliance was similar between morning (mean 84.2%, SD 13.1%) and evening (mean 85.8%, SD 11.2%) prompts (Table 2), with rates ranging from 17% to 97% among the participants (Figure S1 in Multimedia Appendix 1*).*

**Table 2.** Mean Ecological momentary assessment (EMA) compliance by prompt type during the 30-day study period (N=9).

EMA compliance measure	Values, mean (SD)
Overall compliance	85 (8.7)
Morning prompts	84.2 (13.1)
Evening prompts	85.8 (11.2)

**Figure 1.**
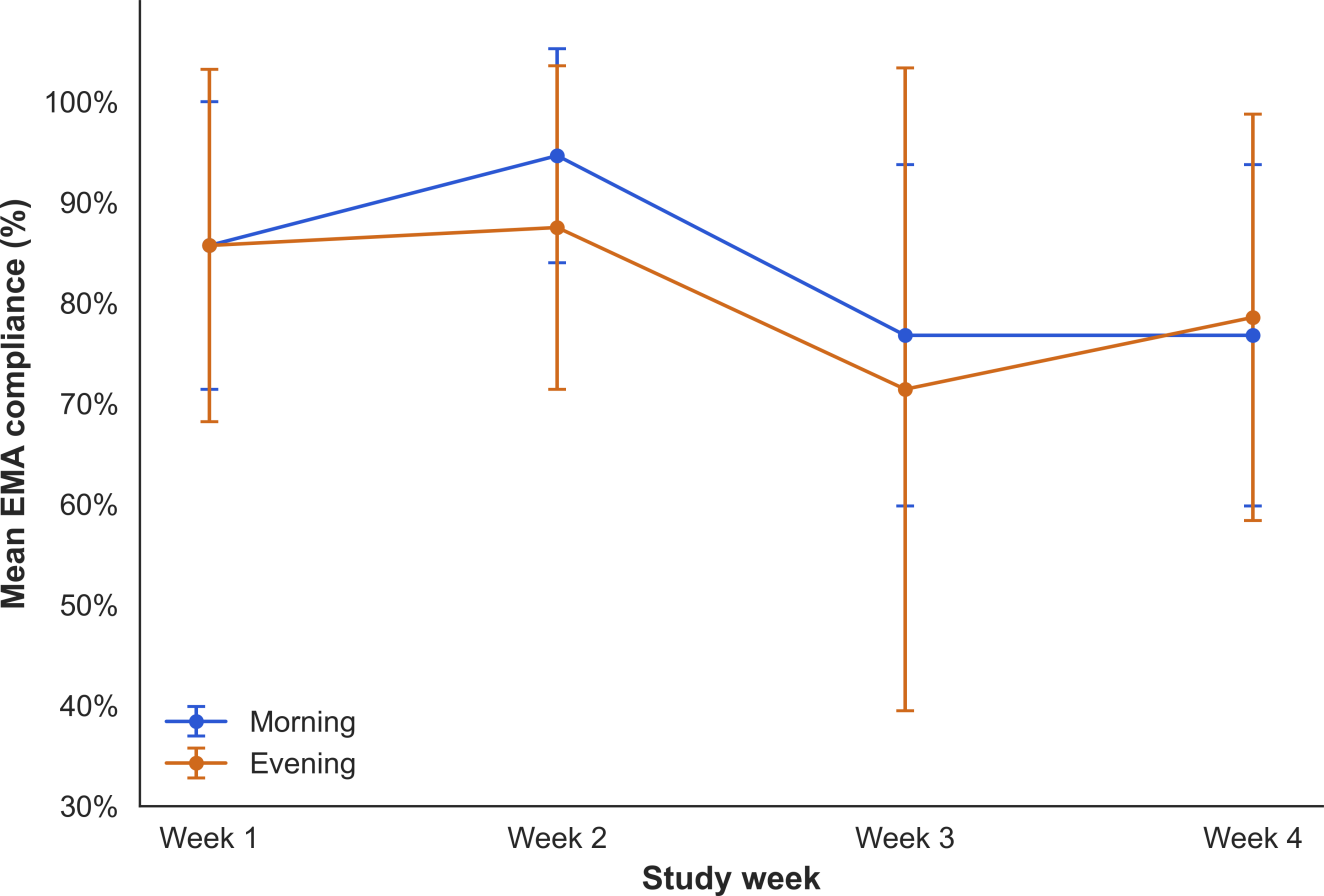
Ecological momentary assessment (EMA) compliance across the 4 weeks of the participation period.

Average EMA compliance varied across participants over the 4 weeks of participation, with similar patterns observed for morning and evening prompts (Figure 1). Morning EMA compliance was highest in week 2 (mean 94.6%, SD 10.6%), followed by week 1 (mean 85.7%, SD 14.3%). Compliance declined in week 3 (mean 76.8%, SD 17.0%) and remained stable in week 4 (mean 76.8%, SD 17.0%). Evening EMA compliance followed a comparable trajectory, peaking in week 2 (mean 87.5%, SD 16.1%) and then in week 1 (mean 85.7%, SD 17.5%). Compliance decreased in week 3 (mean 71.4%, SD 31.9%) and increased in week 4 (mean 78.6%, SD 20.2%).

#### Drug-Related Behavioral Reporting

Weekly reporting patterns for drug-related behaviors across the 30-day study period are presented in Table 3, with participant-level reporting trajectories shown in Figure S2 in Multimedia Appendix 1. Drug craving was reported in 66.7% (274/411) of completed prompts overall, ranging from 58.4% (66/113) to 76.9% (83/108) across study weeks. Plans to use drugs were reported in 61.3% (252/411) of completed prompts overall, ranging from 59.3% (67/113) to 62.5% (55/88) across weeks. Opioid use was reported in all completed EMA prompts across all study weeks. Overdose experiences were reported by 2 participants, with a total of 5 reports across the study period 1.2% (5/411). Naloxone possession remained consistently high throughout the study period, reported in 87.1% (358/411) of completed prompts overall and ranging from 80.4% (82/102) to 94.7% (107/113) across study weeks.

**Table 3.** Weekly reporting patterns of drug-related behaviors across ecological momentary assessment (EMA) prompts.

Behavior	Total reports, n (%)	Week 1, n (%)	Week 2, n (%)	Week 3, n (%)	Week 4, n (%)
Drug craving	274 (66.7)	83 (76.9)	66 (64.7)	59 (67.0)	66 (58.4)
Plan to use drugs	252 (61.3)	67 (62.0)	63 (61.8)	55 (62.5)	67 (59.3)
Opioid use	411 (100)	108 (100.0)	102 (100.0)	88 (100.0)	113 (100.0)
Experienced overdose	5 (1.2)	1 (0.9)	0 (0)	2 (2.3)	2 (1.8)
Carry naloxone	358 (87.1)	90 (83.3)	82 (80.4)	79 (89.8)	107 (94.7)

### Acceptability

**Figure 2.**
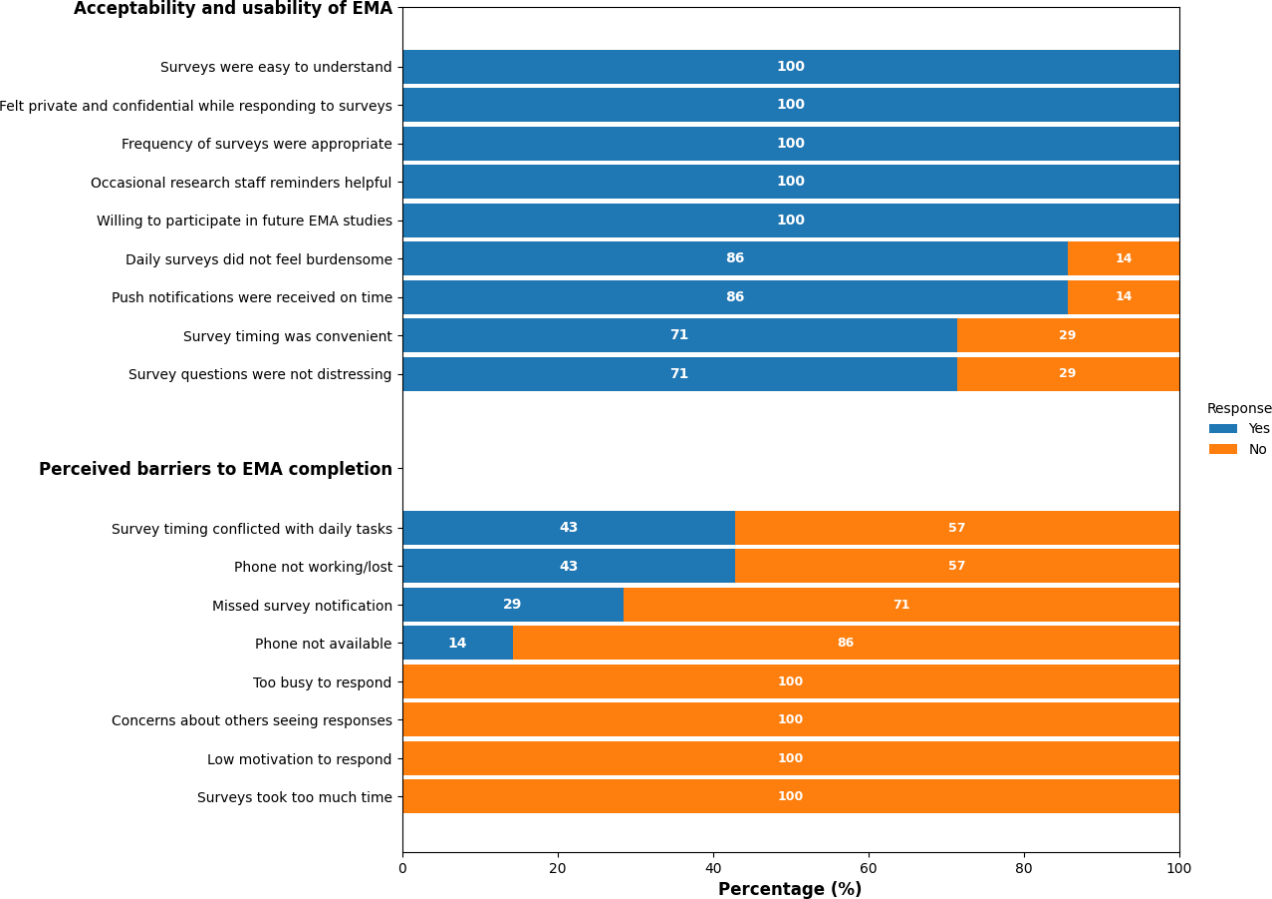
Participant-reported acceptability, usability, and perceived barriers to ecological momentary assessment (EMA; n=7).

Overall, the acceptability and usability of the EMA protocol were high (Figure 2). All participants reported that the EMA prompts were easy to understand, felt private and confidential, and that the survey frequency was appropriate. All participants also indicated willingness to participate in future EMA studies and found occasional reminders from research staff helpful. Most participants (6/7, 86%) reported receiving push notifications on time and indicated that daily EMA prompts did not feel burdensome. EMA timing was considered convenient by 71% (5/7) of participants, and 71% (5/7) also reported that the survey questions were not distressing.

Participants also reported a few barriers to EMA completion. EMA timing conflicts with daily tasks and phone-related issues (eg, phone not working or lost) were each reported by 43% (3/7) of participants. Missed EMA notifications were reported by 29% (2/7) of participants, and lack of phone availability at the time of the EMA was reported by 14% (1/7) of participants. None of the participants reported low motivation, concerns about privacy, or prompt length as barriers to completing the EMA.

### Qualitative Findings

Seven participants completed the one-on-one semistructured exit interview. Thematic analysis yielded five themes that reflected the participants’ experiences with the EMA study: (1) ease of use and integration into daily routines, (2) self-awareness and reflection related to substance use, (3) perceived accountability through self-monitoring, (4) emotional discomfort related to substance use reflection, and (5) situational and technical barriers to EMA prompt completion.

### Ease of Use and Integration Into Daily Routines

Five participants described the EMA prompts as easy to complete and manageable within their daily routines. Participants mentioned that the prompts were brief and delivered at predictable times, which supported routine completion without being perceived as intrusive:

*It was very convenient, because it was two times a day in the morning and at night, so it wasn’t like bugging me or anything.*

[P7]

*I already knew what time they were gonna come around, so I kind of prepared to be ready to do it.*

[P6]

### Self-Awareness and Reflection Related to Substance Use

Four participants reported that repeated reporting increased their awareness of their substance use patterns and daily behaviors. Some participants noted that completing the EMA prompts encouraged them to reflect on their cravings, intentions to use substances, and substance use experiences throughout the day:

*It showed me that it is true that the first hit makes me crave more… it really showed me that I needed some help.*

[P6]

*A lot of the time, I wanted to use drugs, but I didn’t plan to, and I didn’t actually do it.*

[P2]

### Perceived Accountability Through Self-Monitoring

Two participants described how regular EMA participation created a sense of accountability, structure, or engagement in their daily routines, even without any intervention or direct interaction with the study staff:

*It felt good… I looked forward to doing it [EMA prompt] twice a day.*

[P1]

*It kept me on my toes… like an assignment or a job, you stay on schedule.*

[P6]

### Emotional Discomfort Related to Substance Use Reflection

Two participants described experiencing emotional discomfort when reflecting on their substance use through repeated EMA reporting. These experiences included feelings of guilt, sadness, or emotional distress associated with reporting substance use behaviors:

*It kinda made me feel a little bit guilty when I complete it [EMA prompt] and report drugs use.*

[P8]

*It sometimes makes you feel sad and not so good about yourself… it’s a hard reality to accept.*

[P4]

### Situational and Technical Barriers

Two participants described situational or technical barriers that occasionally interfered with EMA prompt completion, including oversleeping, low phone battery, and missed alerts:

*When I overslept, and I woke up, and I missed the survey, which was challenging.*

[P2]

*There were a couple of times that my phone was dead and the notification didn’t come through, so I forgot about it.*

[P4]

## Discussion

### Principal Findings

In this study, we explored the feasibility and acceptability of a 30-day smartphone-based EMA protocol among people who use opioids. Recruitment through the SSP was successful and represented an important strength of this study. Overall, 70% of the participants completed the study and follow-up interviews. EMA completion rates were high, with an overall compliance rate of 85%, and similar response rates for morning and evening prompts. Participants regularly reported opioid use, cravings, overdose experiences, and carrying naloxone through EMA, suggesting that twice-daily monitoring of opioid-related behaviors is feasible in community settings.

The EMA compliance rate observed in this study was higher than the pooled compliance rate reported in a meta-analysis of 126 effect sizes from 19,431 participants among EMA studies of people who use substances (75.1%, 95% CI 72.4%-77.7%) [23]. Prior EMA studies among people who use opioids have largely focused on momentary predictors of drug use, such as craving, mood, and stress, rather than on overdose-related experiences or harm-reduction behaviors. Compliance remained generally stable across morning and evening prompts and throughout the 30-day study period, with a modest decline in the third week—a pattern reported in other EMA studies and often attributed to participant fatigue [23-25]. In our study, compliance increased again during the final week, suggesting that participants were able to integrate the EMAs into their daily routines. Predictable EMA prompt timing, brief questionnaires, and regular reminders from the research staff may have contributed to these high response rates. Prior EMA studies often included larger samples and longer study durations, which may partly explain the differences in compliance [17,18,37]. In this context, the high compliance observed in our study may have been influenced by the small sample size, short study duration, and participants’ relatively high levels of education and digital literacy [23,38,39].

Importantly, participants reported their drug use behavior and experiences related to overdose and carrying naloxone through daily EMA prompts. Although only a small number of overdose events were reported during the study period, participants reported carrying naloxone on every occasion when an overdose was reported. However, the study did not assess whether naloxone was administered during these overdose events or whether it was used for reversal. These findings suggest that EMA may be a useful approach for capturing timely information on overdose experiences and harm-reduction responses. Prior EMA-based research among people who use opioids has focused on momentary predictors of drug use, such as craving, mood, pain, and contextual factors, but did not assess overdose experiences or whether participants were carrying naloxone [17,18,40-42]. While preliminary, our findings suggest that EMA may also be useful for capturing overdose-related experiences and harm-reduction behaviors in daily life. Such information may help improve the understanding of the circumstances surrounding overdose events and may inform future real-time prevention and intervention strategies.

Despite disclosing minimal barriers (EMA prompt timing, unavailability of phone, and missed prompt notifications), participants reported that overall participation was acceptable, minimally burdensome, and did not interfere with their daily activities. In similar EMA studies [43,44], privacy and confidentiality were reported as major barriers; however, none of the participants in our study raised these concerns. Participants further reported an overall positive experience, with all indicating that they would participate in a similar study in the future. In real-world settings, it would be helpful to consider the barriers they faced, such as prompt timing, missed push notifications, and the emotional discomfort associated with some questions. Fixed notifications, individualized prompt timing, and participant-friendly question wording may help reduce participant burden and improve engagement when EMA protocols are developed using a user-centered approach. In addition, providing participants with personalized feedback based on EMA data may be more impactful [45], which could motivate them to remain engaged for a longer period. Such EMA data may also support the development of mobile health–based just-in-time adaptive interventions, where timely information on drug craving, planned use, or contextual risk factors could be used to predict periods of elevated overdose risk and deliver timely harm-reduction support, such as reminders to carry and use naloxone [46,47]. However, further research is needed to determine how such approaches can be effectively implemented in real-world settings.

### Limitations

Several limitations should be considered when interpreting the findings of this study. First, the sample size was very small, and participants were recruited through a single SSP in New Haven, which limits the stability of the findings and restricts generalizability to the wider opioid-using population. Individuals engaged with harm reduction services may differ from those who are not connected to such programs, including having greater access to harm-reduction resources, higher overdose awareness, and more frequent engagement with health services, potentially limiting the generalizability of the findings to broader populations of people who use opioids. Second, the study sample included individuals with relatively high digital health literacy and smartphone access, which may not reflect the experiences of people who use opioids with lower or more unstable access to digital technologies due to social and structural vulnerabilities, such as housing instability and poverty. Third, EMA items, along with the poststudy acceptability questionnaire, were adapted and were not formally validated, which may limit comparability with other studies. Fourth, all EMA responses and poststudy assessment measures were self-reported and may have been influenced by social desirability or participant fatigue, potentially leading to overreporting of positive experiences and underreporting of barriers. Fifth, although participants reported carrying naloxone, the study did not assess whether naloxone was administered during overdose events. Sixth, the 30-day EMA protocol does not capture long-term adherence or engagement beyond the short monitoring period. Finally, participants received compensation for completing EMA prompts, which may have influenced response rates and engagement observed in this pilot study and may not reflect participation in real-world settings, where similar incentives may not be available.

### Conclusions

Our findings suggest that a smartphone-based EMA protocol is feasible and acceptable among people who use opioids. Participants demonstrated high compliance with twice-daily EMA prompts and reported opioid use, overdose experiences, and naloxone possession. These findings highlight the potential of EMA to capture day-to-day patterns of substance use and overdose-related behavior. Future research should examine how EMA data can be leveraged to inform adaptive interventions, such as just-in-time adaptive interventions, to reduce the risk of overdose. Larger and longer-term studies are needed to further evaluate EMA for monitoring opioid use, supporting harm-reduction strategies, and understanding broader aspects of daily functioning and well-being among people who use opioids.

## Supplementary material

10.2196/95655 Multimedia Appendix 1 Interview guide and supplementary figures with daily ecological momentary assessment (EMA) completion and reported drug-related behaviors.

## Abbreviations

EMA: ecological momentary assessment
NHSSP: New Haven Syringe Services Program
SSP: Syringe Service Program
