# Supplementary material for: Smartphone-Based Ecological Momentary Assessment to Monitor Opioid Use and Overdose Among People Who Use Opioids: Prospective Observational Feasibility Study
Source: JMIR Hum Factors. 2026 Jun 29;13:e95655. doi: 10.2196/95655 (PMC13313411; doi:10.2196/95655)
Supplement: Multimedia Appendix 1 [file humanfactors-v13-e95655-s001.docx]

**Supplementary File 1:** Interview Guide

1. Overall, how did you feel answering the surveys for 30 days?
   *Probes:*
   1. What was that experience like for you (good, distressing, or time-consuming)?
   2. Did answering the daily questions lead you to notice or reflect on anything about yourself?
2. What barriers or challenges, if any, did you face when filling out the daily surveys?
3. Could you describe a moment when completing a daily survey was challenging?

*Probe:*

- 1. What happened during that situation?

1. How might we improve the survey process to make it easier to complete?

*Probe:*

- 1. Are there any changes to the app, question format, or timing that would make participation easier?

1. How did it feel to respond to questions about drug use?
2. Do you think taking part in this study influenced how you think about drug use in any way?

*Probes:*

- 1. What are your current thoughts about it?
  2. Did answering these questions lead you to think about it differently (app, question format, or timing) in any way?

**Supplementary Figure 1**


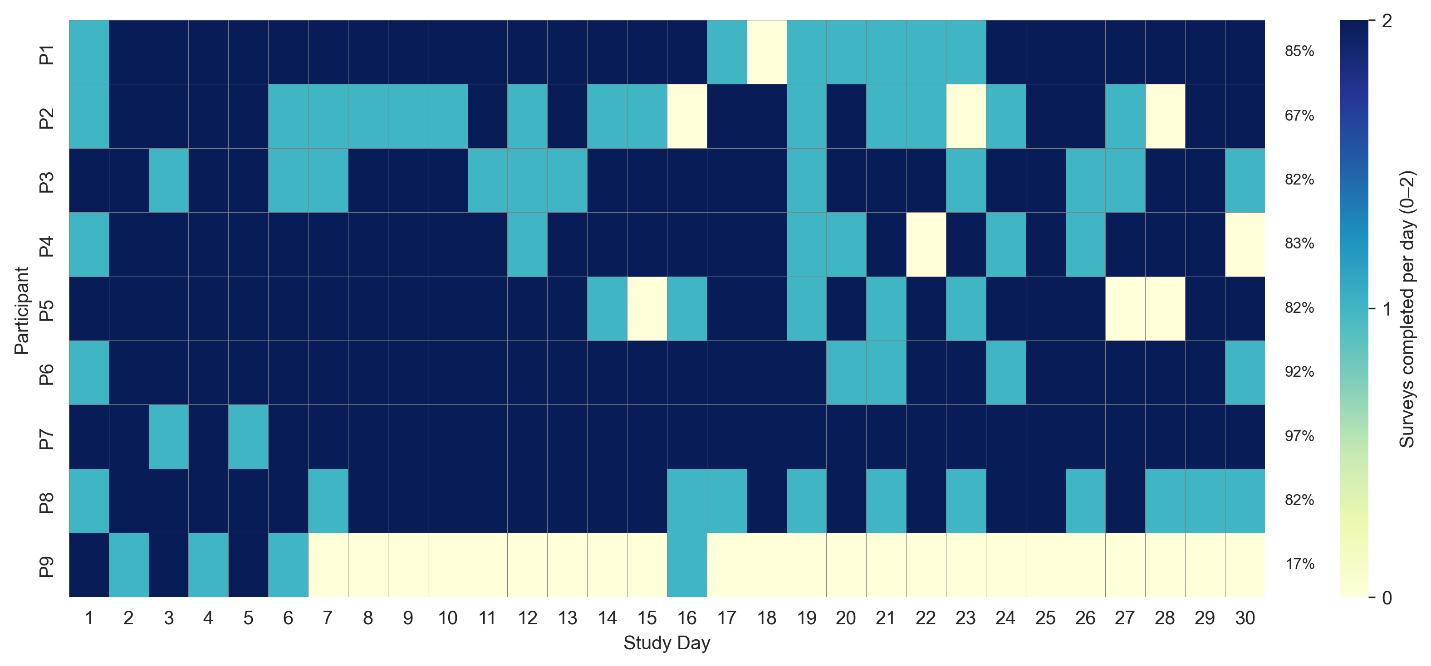


Supplementary Figure 1. Heatmap of daily EMA completion by participant across the 30-day study

**Supplementary Figure 2**


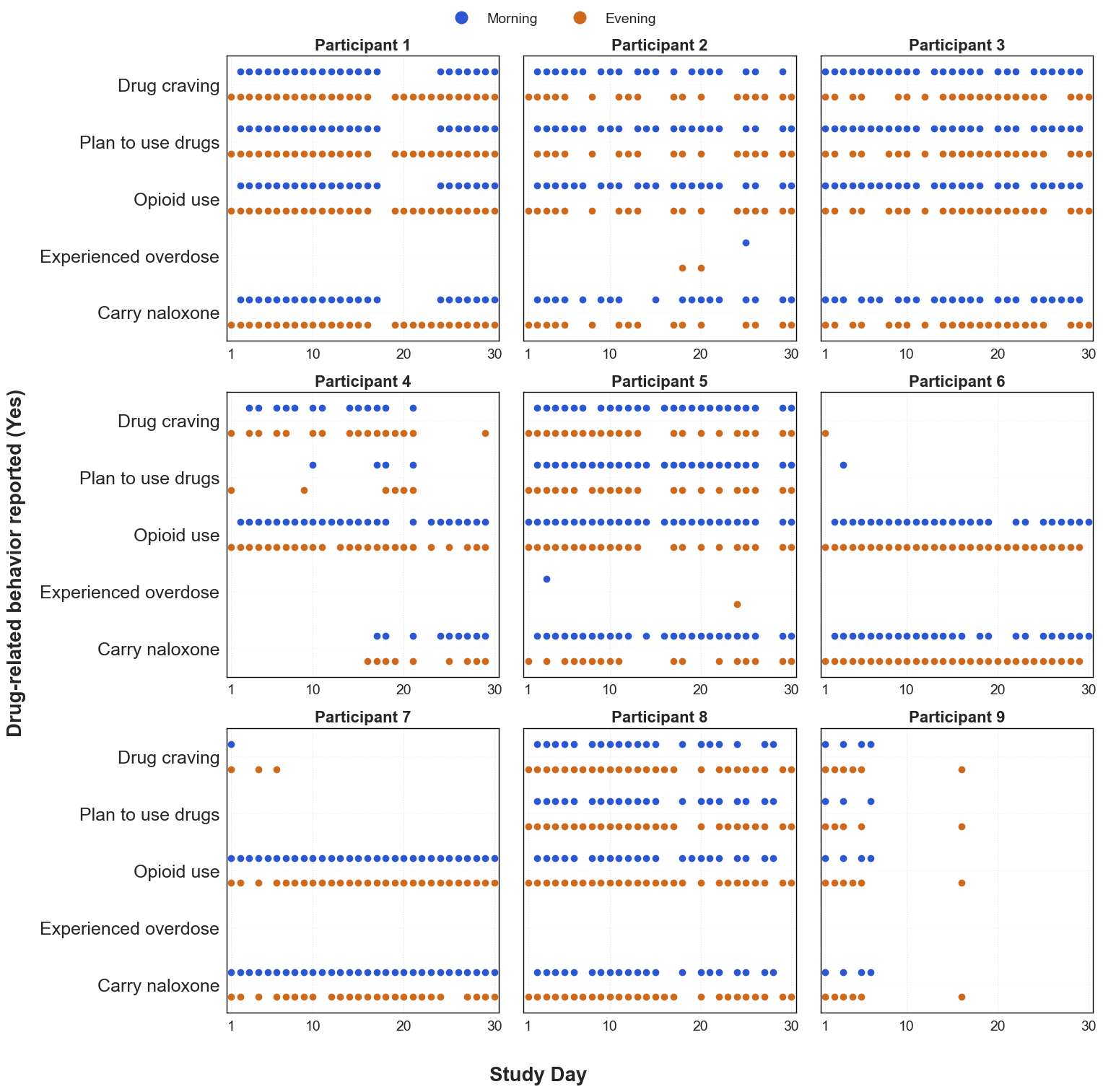


Supplementary Figure 2. Participants reporting drug-related behaviors via morning and evening EMA prompts
